# *Capsicum carassense* (Solanaceae), a new species from the Brazilian Atlantic Forest

**DOI:** 10.3897/phytokeys.140.47071

**Published:** 2020-03-04

**Authors:** Gloria E. Barboza, Luciano de Bem Bianchetti, João Renato Stehmann

**Affiliations:** 1 Instituto Multidisciplinario de Biología Vegetal (IMBIV-CONICET) and Department of Pharmacy, Chemical Science Faculty, University of Córdoba, Casilla de Correo 495, 5000 Córdoba, Argentina University of Córdoba Córdoba Argentina; 2 Empresa Brasileira de Pesquisa Agropecuária – Centro Nacional de Pesquisa de Recursos Genéticos e Biotecnologia (EMBRAPA – Recursos Genéticos e Biotecnologia), PqEB Parque Estação Biológica, Av. W/5 final, Brasília-DF, CEP 70770–917, Caixa Postal 02372, Brasil Centro Nacional de Pesquisa de Recursos Genéticos e Biotecnologia Brasília Brazil; 3 Departamento de Botânica, Instituto de Ciências Biológicas, Universidade Federal de Minas Gerais, Avenida Antônio Carlos, 6627, 31270–901, Belo Horizonte, Minas Gerais, Brazil Universidade Federal de Minas Gerais Belo Horizonte Brazil

**Keywords:** Brazil, *
Capsicum
*, Minas Gerais, new species, taxonomy

## Abstract

*Capsicum carassense* Barboza & Bianchetti **sp. nov.**, a species from mid-elevation of the Atlantic Forest (Minas Gerais, Brazil) is described and illustrated. This endemic new species is distinguished from the most similar *C. mirabile* Mart. by its moderate to dense general pubescence, narrowly elliptic leaves and larger calyx appendages and corollas. A key for the native Brazilian species of *Capsicum* growing in the state of Minas Gerais is also provided.

## Introduction

*Capsicum* L. (Capsiceae, Solanaceae) comprises ca. 41 species of mostly shrubs or subshrubs with axillary sessile inflorescences, truncate calyces or appendages borne below the margin, corollas usually stellate or campanulate and variously coloured and usually pungent fruits (Hunziker 2001, Barboza et al. 2016). Some species have pharmacological properties (Papoiu and Yosipovitch 2010, Al-Snafi 2015, Parvez 2017) and several constitute major vegetable and spice crops worldwide (Jarret et al. 2019), mainly due to the presence of compounds unique to *Capsicum*, the capsaicinoids, which are responsible for the pungency of the fruits. The genus is native to tropical and temperate Central and South America, Mexico and the West Indies (Barboza et al. 2019), with five species widely cultivated throughout Europe, the southern United States, Africa, India, China and South America (Pickersgill 1997, Basu and De 2003, Libreros et al. 2014, Scaldaferro et al. 2018).

There are two main centres of diversity for *Capsicum*, the Andes and Brazil (Barboza et al. in prep.). For both centres, new species have been described in recent years (Barboza and Bianchetti 2005, Nee et al. 2006, Barboza 2011, Barboza et al. 2011, Barboza et al. 2019). Brazil is an extensive country that hosts the most diverse flora within the Americas (Ulloa Ulloa et al. 2017, BFG 2018) and *Capsicum* has 50% of its species growing in its territory. Additionally, Brazil’s Atlantic Forest is the fourth leading hotspot in terms of endemic plants (2.7% of global total, Myers et al. 2000), where ten *Capsicum* species are endemic (Barboza et al. 2011), some with very restricted distributions (e.g. *C. friburgense* Bianchetti & Barboza and *C. hunzikerianum* Barboza & Bianchetti).

While working with the genus on the Flora do Brasil 2020 project, we revised several Brazilian herbaria and visited the Serra do Caraça, a historical natural reserve with remnants of semi-deciduous montane forest in the Iron Quadrangle, in Minas Gerais state. There, we found some populations that were recognised at first glance as belonging to *C. mirabile* Mart., a species that typically occurs in montane rainforests in south-eastern Brazil. *Capsicum* species from different regions of the Atlantic forest were included in the broad molecular phylogeny of the genus, based on nuclear and chloroplast markers (Carrizo García et al. 2016). The new species, denoted as Capsicum aff. mirabile in Carrizo García et al. (2016) was not sister to the *C. mirabile* accession included in these analyses, suggesting that it may be an undescribed taxon. Then, we reviewed in detail the morphology of the *Capsicum* species of the Brazilian interior and coastal forests and determined that the populations from Serra do Caraça and surroundings represent a distinct species of *C. mirabile*. Here, we describe and illustrate this new species and provide comments on taxonomy, ecology and conservation, taking into account its occurrence in a region heavily threatened by mining activities.

## Material and methods

The description is based on observations and data taken from specimens collected in the field between 1986 to 2019, mainly in Serra do Caraça (Minas Gerais, Brazil) and examination of herbarium specimens from 13 herbaria (BHCB, BHZB, BM, CEN, CORD, ESA, JPB, M, MBM, RB, SP, UEC and UT). Specimens have been accessed *in situ* or through digital images via INCT Herbário Virtual (http://inct.splink.org.br), Herbário Virtual Reflora (http://reflora.jbrj.gov.br/reflora/herbarioVirtual) or Global Plants (https://plants.jstor.org/) databases. Measurements were made from preserved material in FAA solution (formaldehyde –acetic acid – ethanol) or from living material using a Zeiss Stemi 2000-C stereomicroscope at 6.5–50× magnification. Information on corolla and fruit colour and pungency of fruit was recorded from living material in the fieldwork. Illustrations were made by composite line drawings from preserved material. Photographs were taken during fieldwork by the authors; images were edited using Adobe PhotoshopVR.

To compare this new species with its morphologically similar congener (*C. mirabile*), about 200 specimens of *C. mirabile* were analysed (see Suppl. material 1), using the same methods (fieldwork and examination of living and herbarium collections). To assess *C. carassense* and *C. mirabile* distributions in Minas Gerais, latitude and longitude data indicated on the labels were directly mapped; other localities were georeferenced by searching the locality by GeoLoc tools (https://splink.cria.org.br/geoloc). QuantumGis (QGIS V. 2.18) was used to build the distribution map. Conservation status was assessed using primarily the IUCN criteria B, geographic range in the form of B1 (extent of occurrence, EOO) and B2 (area of occupancy, AOO) (IUCN 2019). The EOO and AOO were calculated using the Geospatial Conservation Assessment Tool, GeoCAT (Bachman et al. 2011) and AOO was based on a defined cell width of 2 km.

## Taxonomic treatment

### Capsicum carassense

Taxon classificationPlantaeSolanalesSolanaceae

Barboza & Bianchetti sp. nov.

FA39E70E-6A27-5F9D-8504-CB0DF8AD4DF2

urn:lsid:ipni.org:names:77206952-1

Figs 1 , 2 , 3

#### Diagnosis.

*Capsicum carassense* is morphologically most similar to *C. mirabile* Mart., but differs in its moderate to dense pubescence, narrowly elliptical to lanceolate leaf blade with acute to obtuse apices, longer calyx appendages (up to 5 mm) and larger corollas (up to 20 mm in diameter).

#### Type.

Brazil. Minas Gerais: Catas Altas, RPPN Serra do Caraça, trilha da gruta de Lourdes, após a capelinha, 20°05'41"S, 43°28'52"W, 1386 m elev., 26 Oct 2014 (fl), *J.R. Stehmann, L.L. Giacomin, G.E. Barboza & S. Knapp 6347* (***holotype*** [two sheets]: BHCB acc.#174038 [BHCB0019940_1!, BHCB0019940_2!]; ***isotypes***: CORD [CORD00006968!], RB [RB 01220059, acc. # 674586!]; MBM).

#### Description.

**Figure 1.**
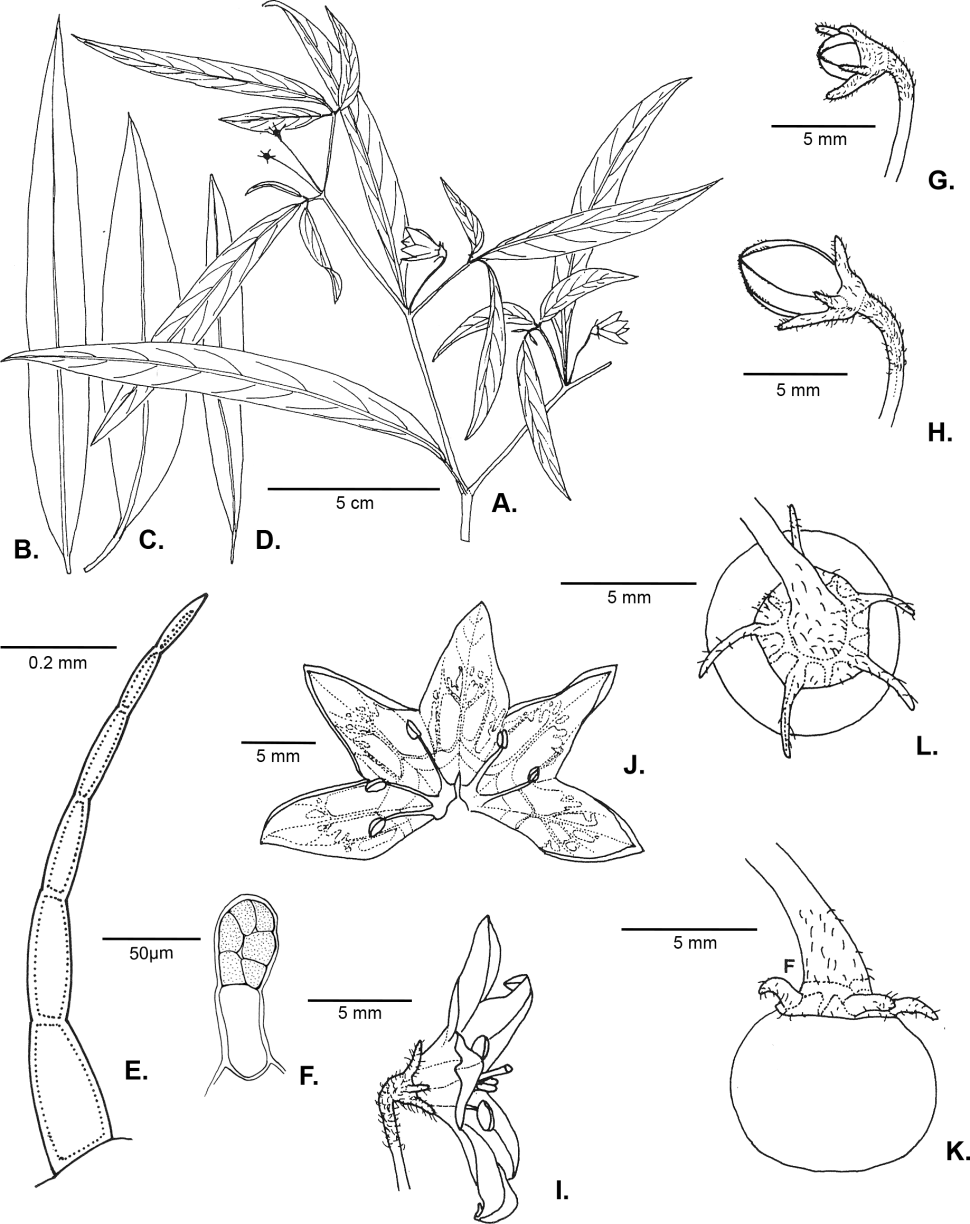
*Capsicum carassense* Barboza & Bianchetti **A** flowering branch **B–D** leaf morphology **E** eglandular trichome of the stem **F** glandular trichome of the calyx **G, H** flower buds in different stages of development **I** flower in anthesis (note the geniculate pedicel) **J** opened corolla **K** fruit **L** fruiting calyx **A–K** from Bianchetti et al. 1364. Drawn by L. Bianchetti.

Shrubs (0.8–) 1–2 (–3) m tall, with the main stem somewhat thick and sparsely branched, the branches dichotomous and spreading horizontally. Stems hollow, angled with ridges; young stems green, striate, moderately to densely pubescent with simple, uncinate and antrorse uniseriate 3–5 (–6)-celled eglandular trichomes 0.2–0.7 mm long, yellowish-brown when dried, the nodes green or purple; bark of older stems brown, pubescent, striate; lenticels absent. Sympodial units difoliate, the leaves geminate or the leaves solitary in the bifurcation of the branches; the leaves of a geminate pair anisophyllous in size. Leaves simple, membranaceous to chartaceous, discolourous, dark green above, paler beneath; adaxial surface moderately pubescent with simple trichomes like those of the stem, especially on the veins; abaxial surface moderately pubescent like the adaxial surface, but with less frequent glandular trichomes with a unicellular stalk and a multicellular head; major leaves with blades 6–16 cm long, 0.9–2.5 cm wide, narrowly elliptic to lanceolate; the major veins 4–6 on each side of midvein, the midvein prominent and the secondary veins obscure, the base attenuate, the margins entire and moderately pubescent, the apex acute to obtuse; petioles 0.2–0.6 cm long, moderately pubescent; the minor leaves 2.9–3.9 cm long, 0.5–0.8 cm wide, narrowly elliptic; the major veins 2–3 (–4) on each side of midvein, the base attenuate, the margins entire, moderately pubescent, the apex obtuse; petioles 0.2–0.4 cm long, moderately pubescent. Inflorescence with the flowers in fascicles of 2–4; pedicels (1.2–) 1.5–2 (–2.2) cm long, slightly angled, erect to oblique, green, geniculate at anthesis, moderately pubescent. Buds ellipsoid, cream with greenish-yellow pigmentation. Flowers 5-merous, all perfect. Calyx 1.2–1.6 mm long, 2.5–3 mm wide, cup-shaped, thin, light green to cream, the margin truncate, pubescent with abundant antrorse curved 3–5-celled eglandular trichomes and sparse short glandular trichomes with a dark elongate, multicellular head and short unicellular stalk (see Fig. 1E, F), the calyx appendages 5, (2.5–) 3–4 (–5) mm long, green, thick, erect, cylindrical, inserted very close to the margin, with the same indument as the calyx tube. Corolla (8–) 10–12 mm long, 13–20 mm in diameter, stellate, thick, with abundant interpetalar tissue, white near the lobe margins and greenish-yellow in the middle and base without, white with 5 purple spots covering the base of the lobes and the throat with a cream centre within, lobed 1/2 or less to the base, the tube 4.5–5 mm long; pubescent in the throat and the base of the lobes with long glandular trichomes with a globose peltate unicellular head and a 2-3-celled stalk inside, the lobes 4.5–6.5 mm long, 5–8 mm wide, broadly triangular to triangular, the tips cucullate, the margins densely pubescent. Stamens subequal; filaments 2.7–3.1 (–4.1) mm long, white, glabrous, inserted on the corolla ca. 1 mm from the base, with inconspicuous auricles; anthers 1.5–1.9 mm long, elliptic, the thecae blue, the pollen whitish-cream. Ovary 1.3–1.5 mm long, ca. 1.2 mm diam., light green, subglobose to ovoid, glabrous; nectary ca. 0.3 mm high, conspicuous; style 4.3–5 (–7) mm long, white, clavate, glabrous; stigma ca. 0.2 mm long, ca. 0.7 mm wide, cream, discoid. Fruit a globose-depressed berry 6–7 mm in diameter, green when immature, yellowish-green when mature, glabrous, pungent, the pericarp hyaline with very long giant cells, the endocarp alveolate; stone cells absent; fruiting pedicels 1.8–2.5 cm long, pendent and slightly curved, slightly angled and widened at the apex; fruiting calyx ca. 4 mm in diameter, persistent, not accrescent, discoid, yellowish-green, the appendages spreading, green, fleshy and cylindrical. Seeds 7–13 per fruit, 3.5–4 mm long, 2.5–3 mm wide, ellipsoidal to reniform, brownish-black to black, the seed coat deeply reticulate, with small spine-like projections. Chromosome number not known.

**Figure 2.**
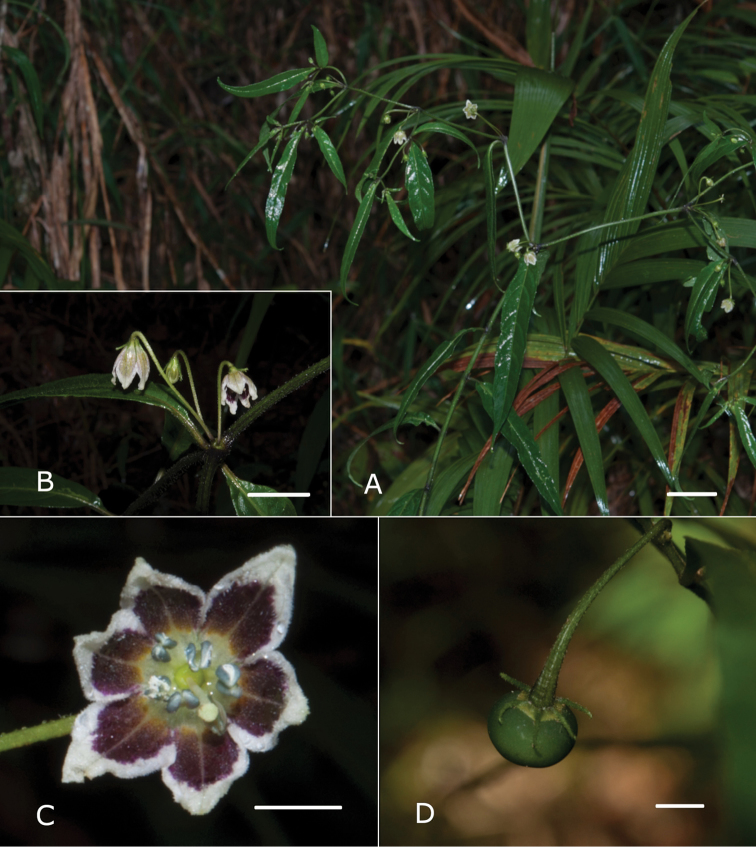
*Capsicum carassense* Barboza & Bianchetti **A** habit, showing the typical lanceolate leaves **B** inflorescence with geniculate pedicels **C** flower, in frontal view **D** globose-depressed fruit **A–C** from Stehmann 6347 **D** from Agra 7268. Photos by J.R. Stehmann. Scale bars: 2 cm (**A**); 1 cm (**B**); 5 mm (**C–D**).

#### Distribution.

**Figure 3.**
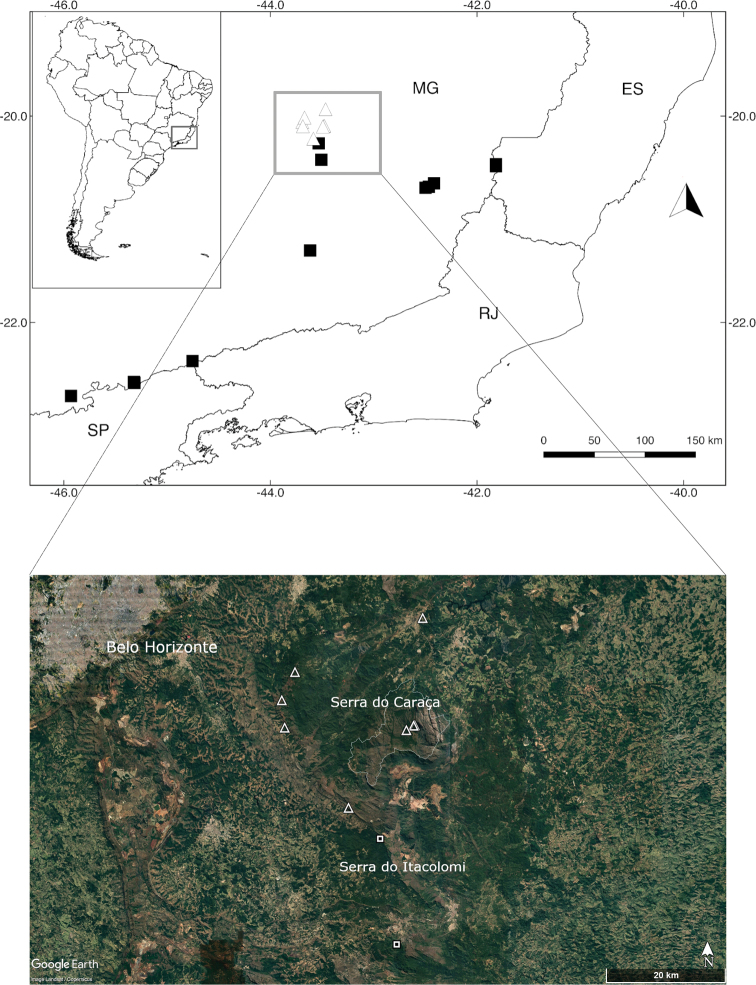
Map showing the distribution of *Capsicum carassense* (triangle) and *C. mirabile* (square) in south-eastern Brazil. Colour map was reproduced and adapted from Google Earth Pro. Abbreviations. MG, Minas Gerais; ES, Espirito Santo; RJ, Rio de Janeiro; SP, São Paulo.

*Capsicum carassense* is endemic to south-eastern Minas Gerais (Fig. 3), growing mainly in the Serra do Caraça and other nearby mountainous areas (Serra do Gandarela, Serra Geral, Serra do Capanema, Serra São Geraldo), between 1000–1390 m elevation.

#### Ecology.

The population studied in the field at Serra do Caraça inhabits the understorey of the semi-deciduous montane Atlantic Forest, in a shaded and moist environment. Information about pollination and dispersal is not yet known.

#### Phenology.

In flower from October to January, also in May; fruiting in December, February and April.

#### Etymology.

The new species is named in allusion to its restricted habitat in the Serra of Caraça and surrounding areas (Minas Gerais, Brazil).

#### Preliminary assessment of conservation status.

Following the IUCN Red List Criteria (IUCN 2019), this species is considered Endangered (EN) B2 a,b (iii, iv). We suggest this category, because of the species’ very restricted geographic distribution (EOO < 483.4 km^2^, AOO < 32 km^2^), as well as the increasingly degraded habitat quality, especially associated with the extensive iron mining activities in the region (see below).

#### Specimens examined.

**Brazil**. **Minas Gerais**: Mun. Catas Altas, Serra do Caraça, near Santa Barbara, trilha a Capela y Gruta de Lourdes, 20°05'39"S, 43°28'45"W, 1430 m elev., 26 Apr 2010 (fr), *M.F. Agra et al. 7268* (BHCB, JPB, RB, UT); Serra do Caraça, ca. 70 km sudeste de Belo Horizonte, próximo ao Mosteiro do Caraça, 17 Nov 1977 (fl), *N.D. da Cruz et al. 6291* (SP, RB, UEC); Barão de Cocais, Parque Natural do Caraça (PNC), a 150 m do monastério, perto da Fonte do Bode, 1250 m elev., 19°56'S, 43°28'W, 22 Apr 1986 (fr), *L. Bianchetti et al. 512* (CEN); Barão de Cocais, PNC, 31 May 1992, *L. Bianchetti et al. 1363, 1364, 1367, 1368* (CEN); PNC, muy cerca del monasterio, en el bosquecillo vecino al Fonte de Bode, ca. 1250 m elev., 21 Apr 1986 (fr), *A.T. Hunziker et al. 25206* (CORD); PNC, muy cerca del monasterio, 1250–1300 m elev., 10 Dec 1986 (fl), *A.T. Hunziker et al. 25256* (CORD, BM); Serra do Caraça, 4 Dec 1999 (fl), *R.C. Mota 106* (BHCB); at the same locality, 18 Dec 2002 (fl), *R.C. Mota 2260* (BHCB); at the same locality, 5 Jan 2005 (fl), *R.C. Mota 2653* (BHCB); RPPN Caraça, Trilha para Tanque Grande, 03 Dec 2013 (fr), *J. Ordones et al. 2229* (BHZB); Serra do Caraça, 1600 m elev., 12 Sep 1990 (fl), *J.R. Stehmann et al. s.n.* (ESA 33757); Serra do Caraça, Tanque Grande, 16 Feb 2012 (fr), *J.R. Stehmann & F.S. Faria 6269* (BHCB); na trilha para o Tanque Grande, 20°06'05"S, 43°29'33"W, 1249 m elev., 26 Oct 2014 (fl), *J.R. Stehmann et al. 6344* (BHCB, RB); Mun. Ouro Preto, Mina da Capanema (Mina da Serra Geral), RPPN Vale, near town of Glaura, trail to Cabeza do Macaco, 20°13'04"S, 43°35'05"W, 1691 m elev., 29 Apr 2011 (fr), *M.F. Agra et al. 7340* (BHCB); estrada da torre-Samarco Mineração-Antonio Pereira, 16 Dec 1996 (fl, fr), *M. Brosehel & J. Craig 406* (RB); RPPN Capanema, 19 Oct 2015 (fl), *M.O. Pivari et al. 2768* (BHCB); Mun. Santa Barbara, Serra de Gandarela/C2, 20°3'24"S, 43°41'28.60"W, 1637 m elev., 26 Nov 2008 (fl), *F.F. Carmo & L.C. Ribeiro 3527* (BHCB); Santa Barbara, 20°00'52"S, 43°40'13"W, 1497 m elev., 17 Dec 2014 (fl), *F.D. Gontijo et al. 589* (BHCB); Rio Acima, Serra do Gandarela, 20°05'52"S, 43°41'12"W, 12 Dec 2011 (fl), *C.V. Vidal & R.L. de Paula 1157* (BHCB); Without municipality: habitat in irriguis lapidosis Serra do S. Geraldo, w/d, *C.F.P. von Martius s.n.* (M 0171537).

#### Discussion.

*Capsicum carassense* belongs to the Atlantic Forest clade (sensu Carrizo García et al. 2016, as Capsicum aff. mirabile). For many years, this species has long been confused with *C. mirabile* in herbaria (Bianchetti 1996, Barboza per. obs.). Both species share similar traits, such as habit, geniculate pedicels at anthesis, number of calyx appendages, the shape and colour of the corolla, colour and pungency of the fruits and blackish seeds. They can be easily distinguished by the indumentum, shape of the major leaf and its length/width ratio, length of the calyx appendages, corolla size and ecology and distribution (see Table 1 for contrasting details).

**Table 1.** Differences between *C. carassense* and *C. mirabile*. Abbreviations. BA: Bahia, ES: Espírito Santo, RJ: Rio de Janeiro, SP: São Paulo, MG: Minas Gerais.

Character	*Capsicum carassense*	*Capsicum mirabile*
Indumentum	Moderately to densely pubescent	Glabrate to sparsely pubescent
Major leaf shape	Narrowly elliptical to lanceolate, apex acute to obtuse	Elliptical to ovate, rarely narrowly elliptical, apex acuminate to long acuminate
Major leaf length/width ratio	(4–) 5–10 (–16)	(2–) 2.5–4 (–4.9)
Buds colour	Cream with greenish-yellow tones	Purple
Calyx appendages	Long appendages (2.8–) 3–4 (–5) mm	Short to long appendages
		(0.4–) 0.5–1.5 (–3) mm
Corolla size	(8–) 10–12 mm long	(6–) 7.5–12 mm long
	13–20 mm in diameter	(9–) 10–13 mm in diameter
Distribution and ecology	Endemic to Serra do Caraça and surrounding areas (MG); mostly in semi-deciduous montane forests	Widely distributed in eastern and southern Brazil (BA, ES, RJ, SP, MG); mostly in dense ombrophilous montane forests

*Capsicum carassense* is a pubescent low shrub with very narrow leaves and large white corollas with purple-spots. The shape and length/width ratio of the leaves of *C. carassense* are very close to the description of C. mirabile var. grandiflorum Sendtn. (Sendtner 1846) but Sendtner also stated that this variety had “floribus majoribus” and “planta glaberrima”. The diameter of the corolla measured in three flowers in the F neg. 2871 of the destroyed varietal holotype (Sellow 209, Herb. Reg. Berolinense) is not more than 1 cm (https://collections-botany.fieldmuseum.org/project/6454), thus these two traits, size of the corolla and lack of pubescence fit with the concept of *C. mirabile* rather than *C. carassense*.

*Capsicum carassense* and *C. mirabile* differ in geographic distribution, with the former inhabiting mostly the understorey of the semi-deciduous montane forests of the southernmost areas of the Espinhaço Range in Minas Gerais, while the latter has a wider distribution (Barboza and Bianchetti 2005), growing mostly along the dense ombrophilous montane forest of south-eastern Brazil, an area characterised by high rainfall and humidity and the absence of a pronounced dry season (Silva Magnago et al. 2007). Both species have a contact zone at the municipalities of Mariana and Ouro Preto, in the Serra do Itacolomi, where *C. carassense*, as well as two other species, *C. mirabile* and *C. villosum* Sendtn., were recorded. There is no information about edaphic preferences for these species, nor possible events of hybridisation in this contact area.

All collections of the new species come from the Iron Quadrangle in Minas Gerais, except one historical Martius specimen at M [M!, photo n° 6522 at F!], collected in the Serra de São Geraldo between Mariana and Presídio de São João Batista (today the municipality of Visconde do Rio Branco). This material, a syntype of *C. mirabile* (Sendtner 1846), was examined when Barboza (2011) lectotypified *C. mirabile*. She stated that “the third syntype […] is unusually pubescent for this species”. Here, we re-examined this specimen housed at M (M–0171537) and concluded that it actually belongs to *C. carassense*.

The relationships amongst the species belonging to the Atlantic Forest clade appear to be fully resolved and an apparent phase of rapid speciation has been suggested for this lineage (Carrizo García et al. 2016). In spite of the morphological similarity between *C. carassense* and *C. mirabile*, these species are not closely related phylogenetically, as *C. carassense* (as Capsicum aff. mirabile in Carrizo García et al. 2016) is not sister to *C. mirabile*, but occurs in a different subclade of the Atlantic Forest clade. In the analysis of Carrizo García et al. (2016), *C. mirabile* appears closest to C. villosum var. muticum Sendtn.

The new species deserves conservation attention, because few populations are known and most of them are distributed in the Iron Quadrangle and associated with remnants of native forests. This area was assessed as priority for conservation in the state of Minas Gerais (Drummond et al. 2005), with high animal and plant diversity and extensive threats, especially from iron mining activities (Jacobi et al. 2011, Salles et al. 2018). The impacts on native vegetation, especially forests, are high because of the activities associated with iron and bauxite mining as the building of dams and urban expansion, all increase deforestation pressures at regional-scale (Sonter et al. 2014).

### Artificial key to the 11 native Brazilian species growing in the state of Minas Gerais (excluding cultivated species)

**Table:** 

1	Calyx without teeth or with 5 very short appendages delimiting a pentagonal outline	**2**
–	Calyx with 5 well-developed appendages, these being 0.4–5 mm long	**6**
2	Pedicels at anthesis non-geniculate, pendent	**3**
–	Pedicels at anthesis geniculate, erect	**5**
3	Leaves elliptic to narrowly elliptic, coriaceous, glabrous; corolla mostly white with purple pigmentation within; fruits greenish-yellow at maturity	***C. pereirae* Barboza & Bianchetti**
–	Leaves elliptic to ovate, membranaceous, glabrescent to moderately pubescent; corolla mostly white or purple with yellowish-green pigmentation within; fruits red at maturity	**4**
4	Inflorescence with (1–) 2–3 (–6) flowers; corolla white with 5 yellowish-green spots at the base of the lobes and throat within; seeds blackish-brown	***C. flexuosum* Sendtn.**
–	Inflorescence with 5–13 (–20 or more) flowers; corolla mostly purple with yellowish-green centre and white margin within; seeds pale yellow	***C. caatingae* Barboza & Agra**
5	Corolla 4.5–6.5 (–8) mm long, with golden spots in the base of the lobes and throat within, purple pigmentation absent; ovules 2 per locule; fruits globose-compressed	***C. campylopodium* Sendtn.**
–	Corolla 7–8 (–10) mm long, with an obvious purple or brownish zone over the greenish-yellow spots within (occasionally purple pigmentation absent); ovules more than 2 per locule; fruits globose or globose-depressed	***C. schottianum* Sendtn.**
6	Corolla rotate, rotate-pentagonal or stellate-rotate; fruiting pedicels always erect, red, usually globose or less frequently ellipsoid; seeds pale yellow	**7**
–	Corolla stellate; fruiting pedicels always pendent, greenish-yellow, globose; seeds brown to black	**8**
7	Corolla mostly white with greenish-yellow spots and white centre, lilac or purple pigmentation absent	**C. baccatum L. var. baccatum**
–	Corolla mostly lilac or purple with greenish-yellow spots and cream centre	***C. praetermissum* Heiser & P.G. Sm.**
8	Pedicels non-geniculate, pendent; major leaves small to medium-sized 2.5–6.5 (–9) cm long	***C. parvifolium* Sendtn.**
–	Pedicels geniculate, erect to oblique; major leaves medium- to large-sized (5–) 6–17 (–25.5) cm long	**9**
9	Plants densely pubescent on stems, petioles, pedicels and sometimes also on the leaf nerves beneath, the trichomes spreading; leaves ovate	***C. villosum* Sendtn.**
–	Plants glabrate to densely pubescent on stems, leaves and pedicels, the trichomes antrorse; leaves elliptic to very narrowly elliptic or lanceolate, less commonly ovate	**10**
10	Plants glabrate to sparsely pubescent; major leaves elliptic to ovate, rarely narrowly elliptic (length/width ratio: (2–) 2.5–4 (–4.9), apex acuminate to long acuminate; calyx appendages (0.4–) 0.5–1.5 (–3) mm; corolla (6–) 7.5–12 mm long, (9–) 10–13 mm in diameter	***C. mirabile* Mart.**
–	Plants moderately to densely pubescent; major leaves narrowly elliptic to lanceolate (length/width ratio: (4–) 5–10 (–16), apex acute to obtuse; calyx appendages (2.8–) 3–4 (–5) mm; corolla (8–) 10–12 mm long, 13–20 mm in diameter	***C. carassense* Barboza & Bianchetti**

## Supplementary Material

XML Treatment for Capsicum carassense

## Supplementary materials

Supplementary material 1 Searchable CSV file of all specimens examined for *C. carassense* and *C. mirabile* Data type: CSV file https://binary.pensoft.net/file/385145This dataset is made available under the Open Database License (http://opendatacommons.org/licenses/odbl/1.0/). The Open Database License (ODbL) is a license agreement intended to allow users to freely share, modify, and use this Dataset while maintaining this same freedom for others, provided that the original source and author(s) are credited.Gloria E. Barboza, Luciano de Bem Bianchetti, João Renato Stehmann
